# Innovation of clockwise osseodensification technique for primary stability in dental implant: a low-density bone cadaveric study

**DOI:** 10.3389/fdmed.2025.1712749

**Published:** 2025-11-12

**Authors:** Pawornwan Rittipakorn, Warisara Ouyyamwongs, Kwanwong Boonpitak

**Affiliations:** 1 Institute of Dentistry, Suranaree University of Technology, Nakornratchasima, Thailand; 2 Institute of Medicine, Suranaree University of Technology, Nakornratchasima, Thailand; 3 Division of Prosthodontics, Faculty of Dentistry, Thammasat University, Pathum Thani, Thailand

**Keywords:** dental implant(s), bone remodeling/regeneration, biomechanics, cranio-maxillofacial surgery, bioengineering, mechanical properties

## Abstract

**Introduction:**

This cadaveric study evaluated the effect of a novel clockwise osseodensification (OD) technique on primary implant stability in low-density bone.

**Materials and methods:**

Forty implants were placed in paired sites of nine formalin-fixed human tibiae, comparing OD (*n* = 20) with standard drilling (SD; *n* = 20). Primary stability was assessed by maximum insertion torque (IT) and implant stability quotient (ISQ). Postoperative bone-implant interface characteristics were examined using cone-beam computed tomography (CBCT), periapical radiography, and synchrotron-based x-ray tomographic microscopy (SR-µCT).

**Results:**

The OD group showed higher mean ISQ (67.5 ± 6.5) and IT (34.0 ± 6.6 Ncm) values than the SD group (62.9 ± 9.3; 29.5 ± 7.6 Ncm, respectively), although these differences were not statistically significant (*p* > 0.05). The results indicate a trend toward improved primary stability with OD (ISQ: *p* = 0.077; IT: *p* = 0.052). A statistically significant moderate positive correlation between IT and ISQ was observed in the OD group (*ρ* = 0.577, *p* = 0.0077) but not in SD (*ρ* = 0.208, *p* = 0.3778), indicating greater predictability of stability outcomes with OD. Radiographic analysis revealed denser peri-implant bone and reduced radiolucency in OD sites, indicating a tendency toward improved bone compaction and closer implant contact. SR-µCT observations qualitatively demonstrated a more condensed trabecular architecture around OD implants compared with SD, consistent with enhanced local bone compaction.

**Discussion:**

These findings indicate that OD produces more consistent stability values and a stronger IT–ISQ relationship than SD, potentially enhancing the reliability of resonance frequency analysis in low-density bone. Unlike conventional counterclockwise OD, Clockwise OD uses densifying burs in the cutting direction at moderate speeds (800 rpm), offering a simpler, less technique-sensitive alternative without sacrificing the benefits of bone condensation. Within the limitations of a cadaveric model, OD demonstrated consistent stability values and a trend toward improved primary mechanical outcomes compared with SD. Further *in vivo* studies are required to confirm these findings and evaluate long-term biological effects.

## 1 Introduction

The success of dental implantation is determined by two critical factors: primary implant stability and subsequent osseointegration. Primary stability plays a pivotal role during the initial healing phase, while osseointegration ensures long-term secondary stability. Achieving adequate primary stability is contingent upon the quality and quantity of the bone at the osteotomy site, as well as the surgical technique employed (1). Inadequate primary stability is a key predictor of early implant failure, compromising the long-term success of the treatment (2). Poor bone quality is associated with implant failure. Low-density bone, classified as D3 and D4, presents a particularly challenging condition for achieving predictable implant stability (3, 4). Low-density bone is most commonly found in the posterior maxilla and in elderly patients, often resulting in insufficient primary stability for dental implants. Accordingly, several studies have reported reduced implant success rates in these anatomical regions when using conventional drilling techniques (5–7).

To address the challenge of low-density bone, several surgical techniques have been developed to enhance primary stability. One common approach is the undersized drilling technique, which has been shown to increase insertion torque and implant survival rates compared to conventional drilling. However, its efficacy remains debated, as some studies report no significant difference (8, 9). Another established method, the osteotome technique, aims to increase bone density through lateral condensation. Despite this benefit, this technique is also associated with potential drawbacks, including excessive pressure leading to bone damage and prolonged marginal bone resorption (10). More recently, the osseodensification (OD) technique was introduced as a novel approach that densifies bone during osteotomy preparation. This process is purported to increase bone density, leading to improved bone-to-implant contact (BIC) and enhanced primary stability (11, 12).

The standard osseodensification protocol involves a specific drilling sequence using specially designed burs rotating in a counter-clockwise (CCW) direction, typically at speeds around 1,200 rpm. According to the manufacturer (Versah LLC, Jackson, MI, USA), this non-cutting rotation is intended to facilitate bone condensation into and along the walls of the osteotomy, effectively expanding the site in low-density bone. This process creates a densified layer that has been shown to increase insertion torque values (ITV) and the implant stability quotient (ISQ) (13). However, this standard CCW technique has several notable limitations. Firstly, its success is highly technique-sensitive because the densifying burs are designed with reverse-cutting flutes that can actively remove bone, contrary to the non-cutting claim (14). This cutting action can lead to increased biomechanical stress, potentially causing shallower implant placement. Secondly, the application of excessive pressure risks permanent plastic deformation of the bone tissue (15). Furthermore, the protocol has specific clinical constraints: it is primarily indicated for cancellous bone with a minimum thickness of 2 mm and requires a modified approach for dense bone (Type D1 or D2), making its outcome highly dependent on clinician skill (11). These challenges highlight the need for alternative or simplified protocols.

To address these limitations, this study explores a clockwise osseodensification (OD) protocol. It is hypothesized that using the specialized burs in the clockwise (CW) direction at a lower rotational speed (800 rpm) can promote lateral bone condensation rather than material removal. In this orientation, the bur's non-sharp flute geometry and negative rake angle generate compressive forces that push trabecular bone laterally against the osteotomy wall, allowing controlled compaction instead of cutting. This mechanical action is expected to reduce local stress and enhance bone density, potentially offering a simpler and less technique-sensitive alternative while maintaining the fundamental principle of osseodensification. This concept has not been thoroughly investigated, and its effect on primary implant stability in low-density bone remains unknown. Therefore, the aim of this cadaveric study is to compare the primary stability of dental implants placed using a clockwise osseodensification technique vs. a conventional drilling technique in a low-density bone model.

## 2 Materials and methods

### 2.1 Specimen selection and preparation

This study was approved by the Human Research Ethics Committee of Suranaree University of Technology (HREC-SUT; approval number: EC-67-0168). Nine formalin-fixed human tibiae were harvested from the lower legs of cadaveric donors. The epiphyseal regions of the tibiae were selected for this study. All remaining soft tissues were meticulously removed from the bone surfaces.

### 2.2 Study design and surgical protocol

A total of forty Osstem Taper TS III implants (4.0 mm diameter, 10.0 mm length; Osstem®, Korea) were placed in this study. The 40 implant sites were organized into two groups (*n* = 20 per group): a Standard Drilling technique (SD) group and a Clockwise Osseodensification technique (OD) group. To minimize inter-specimen variability, a paired-site design was used. The 40 sites were arranged as 20 pairs within the same tibial epiphysis, with each pair comprising two adjacent locations spaced at least 5 mm apart. This allowed for a direct, within-cadaver comparison between the two protocols.

All surgical procedures were performed by a single calibrated author using a surgical motor (Surgic Pro, NSK®, Japan) with constant 0.9% normal saline irrigation. The final osteotomy dimension for all sites was 4.0 mm in diameter and 10.0 mm in length. Countersinking was not performed. For the SD group, the osteotomy was prepared following the manufacturer's instructions. The site was sequentially enlarged using drills of 2.2, 3.5, and 4.0 mm in diameter to a depth of 10 mm at a speed of 800 rpm. For the Clockwise OD group, a bone compaction kit (Osstem®, Korea) was used. The osteotomy was prepared with clockwise-rotating densifying burs of 2.2, 3.5, and 4.0 mm in diameter to a depth of 10 mm, also at 800 rpm. Following osteotomy preparation, all 40 implants were inserted using the surgical motor with a torque set 20 Ncm and then gradually increase to 30, 35 and 40 Ncm until implant seat in the position.

### 2.3 Primary stability assessment

Implants were placed in two designated groups: the SD group (*n* = 20) and the OD group (*n* = 20). A surgical motor (NSK, Tokyo, Japan) was used for insertion at a final speed of 30 revolutions per minute (rpm). The maximum insertion torque (IT) was limited to 40 Ncm, and the final IT value for each implant was recorded. Immediately following implant placement, primary stability was evaluated by measuring the Implant Stability Quotient (ISQ) with a resonance frequency analysis (RFA) device (Osstell ISQ, Gothenburg, Sweden). For each implant, two measurements were taken from four distinct surfaces: mesial, distal, buccal, and lingual. The final ISQ value for each implant was calculated as the mean of all recorded measurements.

### 2.4 Cone-beam computed tomography (CBCT) and radiographic evaluation

Following implant placement, postoperative imaging was performed using high-resolution cone-beam computed tomography (CBCT) and periapical radiography to evaluate the implant sites in both groups. The CBCT scans were acquired with a voxel size of 75 µm, an 80 kVp tube voltage, and a 60 × 60 mm field of view (FOV). Periapical radiographs were taken with a 65 kVp setting and a 0.200-s exposure time. Both imaging modalities utilized the same system (Acteon® X-mind, La Ciotat, France). The acquired images were used for a qualitative comparison of the bone-implant interface between the groups. The assessment focused on morphological features such as the continuity of bone apposition to the implant surface and the presence or absence of interfacial gaps. The evaluation was conducted independently by two calibrated examiners who were blinded to the experimental groups. All radiographic datasets were anonymized and labeled with random numeric codes.

### 2.5 Synchrotron-based x-ray tomographic microscopy (SR-µCT) analysis

For high-resolution three-dimensional analysis, samples from both groups were scanned using synchrotron-based x-ray tomographic microscopy (SR-µCT) at the BL1.2W: x-ray Tomographic Microscopy beamline of the Synchrotron Light Research Institute (SLRI, Nakhon Ratchasima, Thailand). The analysis was conducted using a 13.5 keV monochromatic x-ray beam. Scans were performed to acquire 601 projections over a 180° sample rotation with angular increments of 0.3°, using an exposure time of 0.35 s per projection. This setup yielded an effective pixel size of 3.61 µm with an isotropic voxel resolution. The raw projection data were subsequently reconstructed into 3D volumetric images using the Drishti and Import software packages for visualization and analysis. For qualitative analysis, two representative cross-sectional slices were selected from each reconstructed 3D volumetric dataset: Coronal (central) slice at the midpoint along the implant length, apical slice located approximately 2 mm below the coronal slice. A standardized Region of Interest (ROI) adjacent to the implant interface was defined using consistent anatomical reference points across all specimens. Each image was converted to 8-bit and processed in Fiji (ImageJ, NIH, USA) using thresholding to visually differentiate mineralized bone from void spaces. The resulting images were used for descriptive comparison of peri-implant bone morphology between groups rather than quantitative measurement.

## 3 Statistical analysis

Statistical analysis was performed using RStudio (Version 4.4.1; Posit, PBC, Boston, MA) and GraphPad Prism (Version 10.5.0; GraphPad Software, La Jolla, CA, USA). Continuous data were presented as the mean ± standard deviation (SD). The Shapiro–Wilk test was used to assess the normality of data distribution. A Mann–Whitney *U*-test was performed to compare variables between the OD and SD groups. The relationship between IT and ISQ was evaluated using Spearman's rank correlation coefficient (*ρ*). For all tests, a *p*-value <0.05 was considered statistically significant.

## 4 Results

**Figure 1:**
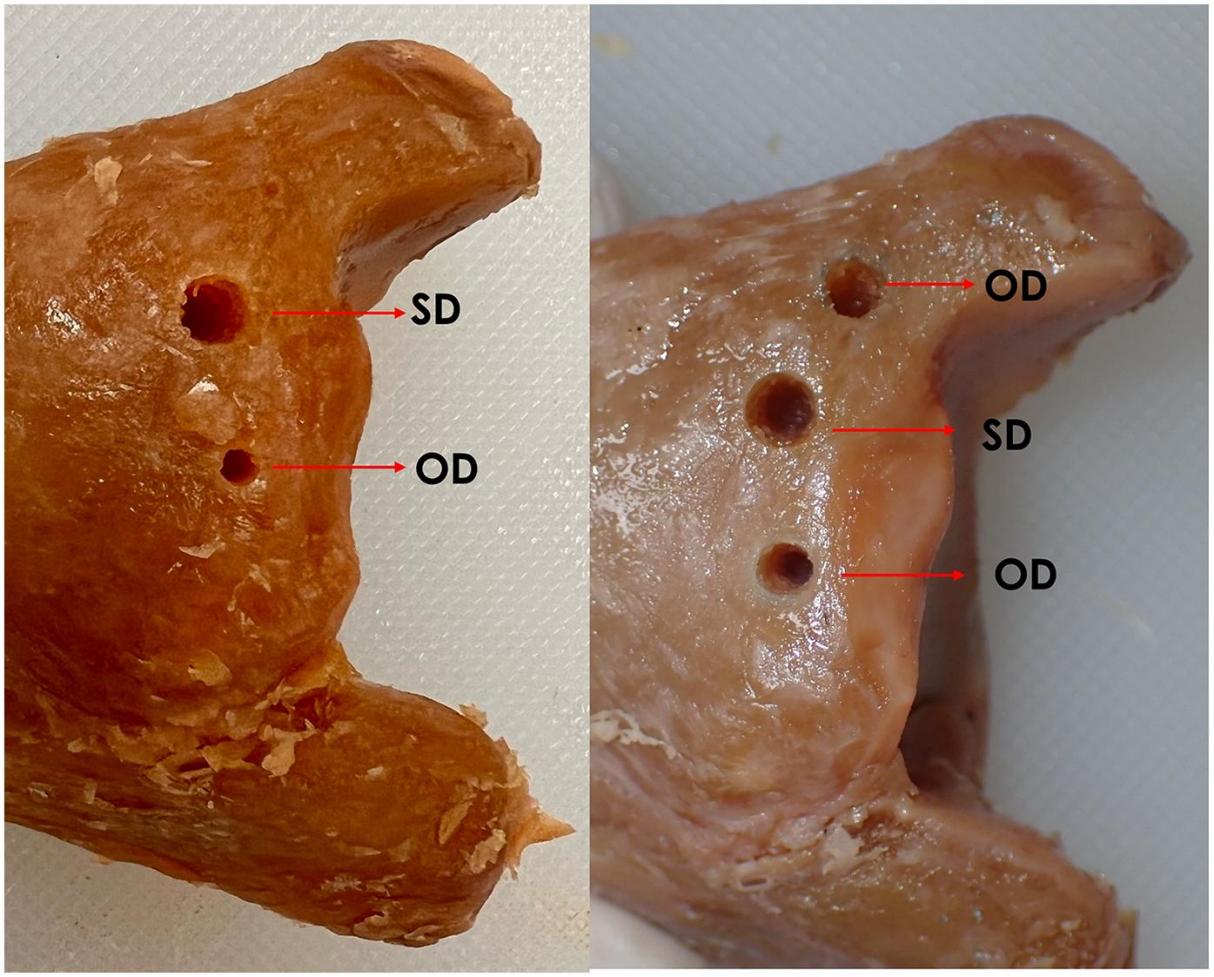
Macroscopic morphology of osteotomy sites prepared using OD and SD techniques in human cadaveric tibiae. The Osseodensification technique site (OD) appears narrower and more condensed, whereas the Standard technique site (OD) shows a wider, less compact morphology.

The samples were sourced from six female and three male donors, with a mean age of 77 ± 9.11 years. A total of 40 implant sites were prepared, equally allocated into the OD and SD groups (*n* = 20 per group). Visual inspection of the osteotomy sites post-drilling revealed a distinct morphological difference between the two techniques, as shown in Figure 1. The osteotomy site prepared with the OD technique was visibly narrower and more constricted compared to the larger, conventionally prepared SD site.

### 4.1 Primary stability outcomes

**Figure 2:**
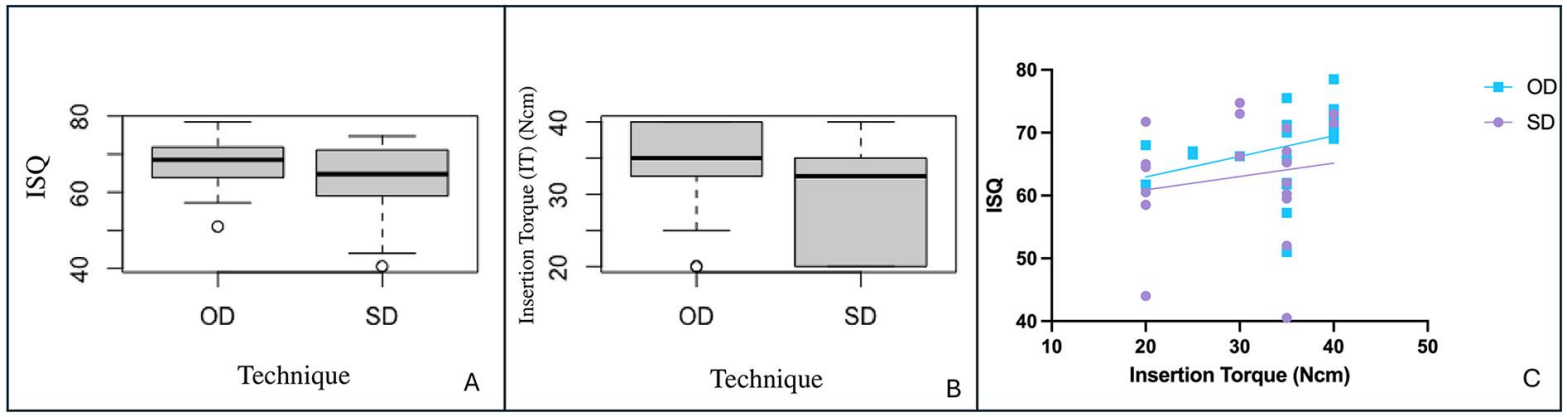
Comparison of primary stability outcomes and their correlation. Box plots show **(A)** Implant Stability Quotient (ISQ) and **(B)** Insertion Torque (IT) for the Osseodensification (OD) and Standard Drilling (SD) groups, with no significant differences observed (*p* > 0.05). **(C)** A scatter plot illustrates a significant positive correlation between IT and ISQ for the OD group (*ρ* = 0.577, *p* = 0.0077), but not for the SD group (*ρ* = 0.208, *p* = 0.3778).

The primary stability outcomes for both techniques are summarized in Table 1 and illustrated in Figures 2A,B. The OD group demonstrated higher mean ISQ and insertion torque values than the SD group (ISQ: 67.5 ± 6.5 vs. 62.9 ± 9.3; IT: 34.0 ± 6.6 Ncm vs. 29.5 ± 7.6 Ncm), although these differences were not statistically significant (*p* = 0.077 and *p* = 0.052, respectively). The boxplot analysis revealed more consistent ISQ outcomes in the OD group, which was characterized by a narrower interquartile range (IQR). The OD group also demonstrated greater consistency in torque values, with a smaller interquartile range and no low outliers observed in the boxplot.

**Table 1:** the mean of the implant stability quotient (ISQ) in OD and SD group.

Outcome	SD (*n* = 20)	OD (*n* = 20)	Difference (95% CI)	*P*-value
Primary outcome				
ISQ	62.925 ± 9.29	67.537 ± 6.45	(−0.52, 9.75)	0.077
Insertion Torque (Ncm)	29.5 ± 7.59	34 ± 6.6	(−0.059, 9.059)	0.052

OD; osseodensification technique, SD; standard technique (*p*-value <0.05).

### 4.2 Correlation between insertion torque and implant stability quotient (ISQ)

The Spearman's rank correlation between ISQ and insertion torque in both groups results indicate the following: correlation Coefficient (*ρ*) = 0.414 which indicates a moderate positive correlation between ISQ and Insertion Torque. When the analysis was stratified by technique group, a distinct difference in this relationship was observed (Figure 2C). The OD group exhibited a statistically significant, moderate positive correlation between the two variables (*ρ* = 0.577, *p* = 0.0077). In contrast, the SD group showed only a weak, non-significant correlation (*ρ* = 0.208, *p* = 0.3778), suggesting no meaningful association between insertion torque and implant stability within this group.

### 4.3 Cone beam CT and x-ray tomographic findings

**Figure 3:**
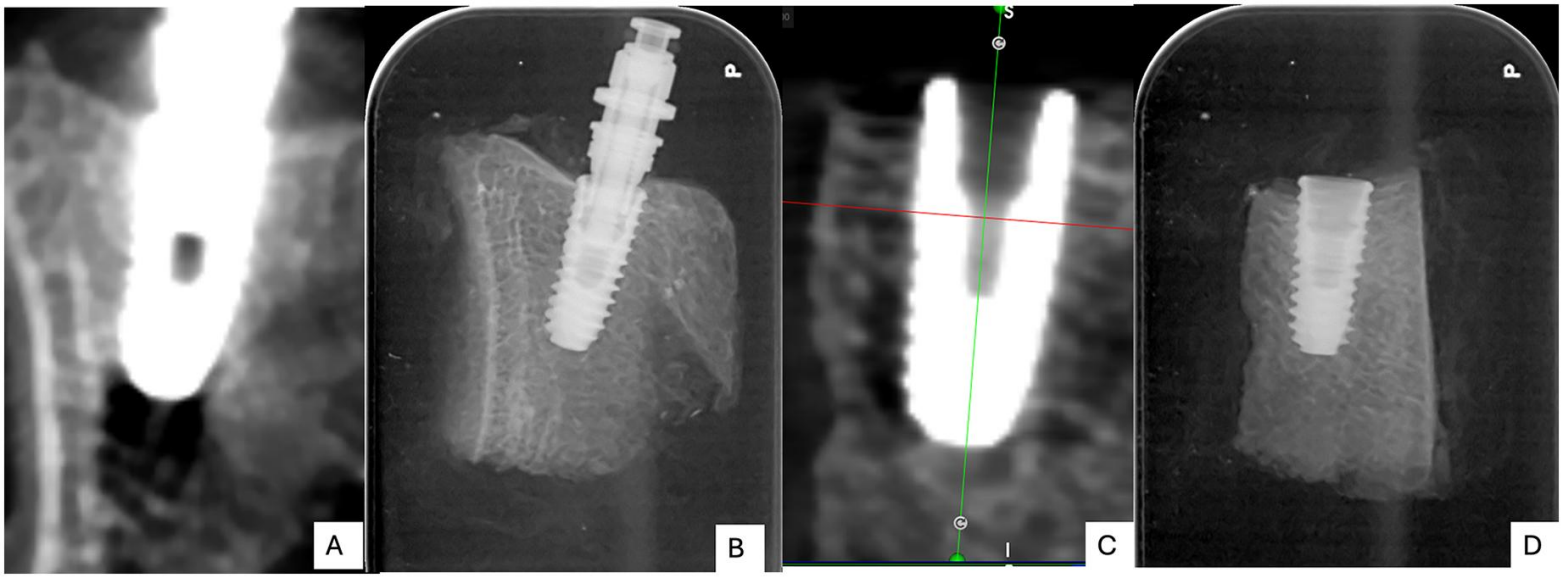
Post-operative cone-beam computed tomography (CBCT) images **(A,C)** and periapical radiographs **(B,D)** of the two groups: osseodensification (OD; **A,B**) and standard drilling (SD; **C,D**).

The Cone beam computed tomography (CBCT) and periapical radiographs revealed differences in the bone–implant interface characteristics between the two groups. In OD group (Figures 3A,B) exhibited greater peri-implant bone densification and enhanced bone-to-implant contact when compared to the SD group (Figures 3C,D). The peri-implant bone appeared more compact and organized, especially along the apical and mid-thread zones. There was minimal radiolucency at the bone–implant interface, indicating improved bone adaptation and initial stability. In contrast, the SD group displayed a less compact trabecular structure surrounding the implant body. The SD group exhibited conventional bone-implant interface patterns typical of standard drilling protocols in Type IV bone, characterized by visible spaces between implant threads and the surrounding bone walls. The trabecular bone pattern remained unchanged from the pre-drilling architecture, confirming the non-condensing nature of standard drilling technique.

### 4.4 Micro-morphological analysis of the bone-implant interface by SR-µCT

**Figure 4:**
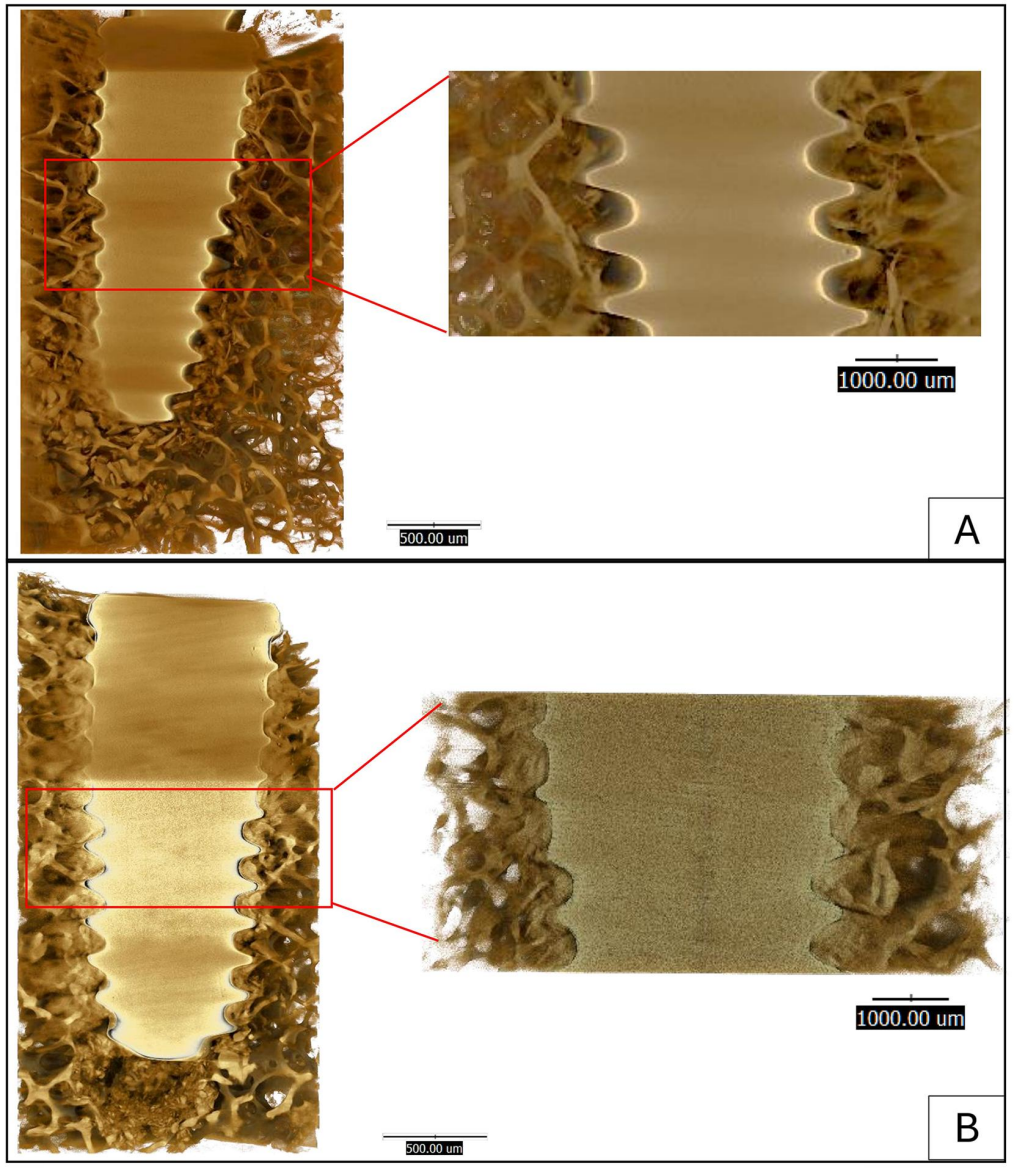
Qualitative and quantitative comparison of the bone-implant interface using synchrotron radiation-based micro-computed tomography (SR-µCT). Representative images show the **(A)** Osseodensification (OD) group and **(B)** the Standard Drilling (SD) group. **(C)** The corresponding quantitative analysis of bone area percent shows no significant difference between the groups (*p* > 0.05).

Qualitative analysis of the high-resolution SR-µCT reconstructions was performed to compare the micro-morphology of the bone-implant interface between the two groups (Figure 4). Visual inspection suggested a trend towards a more condensed bone architecture in the OD group (Figure 4A), with close bone apposition conforming to the implant threads. In contrast, the SD group (Figure 4B) exhibited a more porous peri-implant structure, with visible micro-gaps between the bone and implant surface.

## 5 Discussion

The primary objective of this cadaveric study was to compare the primary stability of dental implants placed in low-density bone using a novel clockwise osseodensification (OD) technique vs. the conventional standard drilling (SD) protocol. The findings from this study indicate a clear trend towards enhanced primary stability with the OD technique when compared with standard drilling. The mean insertion torque in the OD group (34.0 ± 6.6 Ncm) was notably similar to that reported by Mercier et al. (34.9 Ncm), whose study found a statistically significant increase in IT with CCW osseodensification (15). This alignment with previous research, including a systematic review by Padhye et al. which concluded that OD increases IT values (10), strengthens the clinical relevance of our results, despite the *p*-value approaching but not reaching statistical significance (*p* = 0.052). Furthermore, our study adds a crucial observation that the OD technique produced more consistent outcomes, as shown by its narrower interquartile range for both stability metrics. Although the differences in IT and ISQ between OD and SD groups did not reach statistical significance, both parameters demonstrated consistent upward trends toward higher values in the OD technique. Variations in bone density among cadaveric specimens may have reduced the sensitivity to detect smaller differences between groups. Therefore, these findings should be interpreted as clinically relevant trends that warrant validation in larger *in vivo* studies.

A statistically significant moderate positive correlation between IT and ISQ was observed in the OD group (*ρ* = 0.577, *p* = 0.0077), whereas this correlation was absent in the SD group (*ρ* = 0.208, *p* = 0.3778). This stronger IT–ISQ association indicates that the OD technique enhances the predictability of implant stability in low density bone, making resonance frequency analysis (RFA) measurements more reliable for clinical decision-making. In other words, although the absolute increases in IT and ISQ values were small and not statistically significant, the improved correlation reflects a more consistent mechanical response of the bone–implant interface. This improved predictability represents a more clinically meaningful advantage of OD than the minor, non-significant rise in stability values. Previous studies have reported that OD results in significantly higher IT and ISQ values compared with conventional (CD), standard (SD), or undersized drilling (UD) protocols (16, 17). In a human cadaveric study, statistically significant positive correlations were observed between bone density and both IT and ISQ, and notably between IT and ISQ (*r* = 0.853, *p* < 0.001) (18). However, despite OD generally yielding higher IT and ISQ values than SD, the direct correlation between these parameters remains inconsistent across the literature. Reported findings range from weak to moderate positive correlations to no significant relationship, potentially due to differences in study design, bone models (cadaveric, artificial, animal, or human), implant geometry, and specific drilling protocols (19–21). Clinically, achieving predictable primary stability in low-density bone remains a major challenge, particularly in the posterior maxilla. The present findings suggest that the clockwise OD technique may provide a simpler and more forgiving surgical approach, enabling clinicians to achieve stable primary fixation without requiring high rotational speeds or reverse drilling motion. This simplification may help reduce the technique sensitivity associated with conventional counterclockwise OD, improving reproducibility and clinical applicability, particularly for less experienced operators.

Previous studies have primarily investigated osseodensification using CCW burs at high speeds (approximately 1,200 rpm), as recommended by the manufacturer (Versah LLC, Jackson, MI, USA). While effective, the CCW technique is technique-sensitive and may be challenging for inexperienced clinicians. In this study, a simplified clockwise drilling protocol was used, applying densifying burs at 800 rpm in the cutting direction, which may offer a simpler approach while preserving the benefits of bone compaction. Despite the difference in rotation direction, both OD techniques aim to preserve bone integrity by condensing trabecular bone rather than removing it. Our findings are consistent with prior reports demonstrating increased IT and ISQ with OD (11, 22).

The achieved mean IT values in this cadaveric model (34 ± 6.6 Ncm) are comparable to those reported in other studies using low-density bone models (15). Clinically, IT values in posterior maxillary regions typically range from 15 to 50 Ncm, with an average of 28 ± 11.7 Ncm (23), aligning with the present findings. ISQ values generally range between 50 and 80, with successful osseointegration occurring between 57 and 82 (24). These reference points further support the clinical relevance of our observed mean ISQ of 67.5 ± 6.5 in the OD group. While undersized drilling has shown higher IT values, ISQ improvements are often less pronounced (25), reinforcing the importance of evaluating both parameters in tandem.

Radiographic assessments using CBCT and periapical imaging revealed denser peri-implant bone and minimal radiolucency in the OD group, indicating enhanced bone compaction and improved implant-to-bone contact. SR-µCT analysis qualitatively corroborated these findings, showing a denser trabecular structure and closer bone apposition along the implant threads in the OD group compared with the SD group. Because the analysis was based on two-dimensional ROI selection using ImageJ, operator-dependent variability could not be completely eliminated. To minimize this bias, all images were evaluated using standardized anatomical reference points by a single calibrated examiner. The SR-µCT results were interpreted qualitatively to demonstrate micro-morphological trends rather than quantitative measurements. These observations indicate potential microstructural advantages of OD that may contribute to long-term stability. However, it is important to note the inherent limitations of CBCT in quantitative bone-density assessment. Although CBCT is valuable for visualizing peri-implant morphology, gray values (GVs) cannot be reliably converted to Hounsfield units (HUs) as in conventional CT, due to insufficient clinical validation for diagnostic use (26). In this study, CBCT values were used for relative and descriptive comparison within the same device and acquisition protocol, rather than for absolute density measurement. The osseodensification (OD) technique leads to a significant increase in local bone density and bone volume (BV%) by compacting bone debris and leveraging the bone's natural spring-back effect (17, 27). Mechanically, the clockwise rotation of densifying burs is hypothesized to compact bone through controlled plastic deformation and lateral compression, rather than cutting or removing tissue (28). In contrast to the counterclockwise OD protocol, which relies on reverse bur geometry to displace bone chips centrifugally, the clockwise approach allows gradual compression in the cutting direction while maintaining contact with trabecular walls. This promotes localized densification and spring-back of bone, enhancing frictional engagement between the implant and osteotomy walls. Such mechanical compaction likely accounts for the higher and more consistent torque and ISQ values observed in the OD group.

The use of formalin-fixed human cadaveric tibiae provides a reliable preclinical model, with the epiphyseal region representing Type IV bone (<200 HU), analogous to posterior maxillary bone (29). Although formalin fixation alters the organic matrix and reduces elasticity, previous studies confirm that mineral content and macrostructural features remain sufficiently preserved for comparative biomechanical testing (30). Although the epiphyseal region of the tibia exhibits trabecular characteristics and density comparable to Type IV bone, there are anatomical and biomechanical distinctions from the posterior maxilla. In the present study, the tibial epiphysis showed a thicker cortical layer (approximately 1–2 mm) compared with the posterior maxillary bone (around 0.9 mm) (31). These structural differences may influence local stress distribution and the mechanical behavior of bone during implant insertion, thereby limiting direct extrapolation of the current findings to oral conditions. Moreover, the tibiae were not subjected to the functional loading forces that exist in the oral cavity, which could influence long-term stability. Furthermore, the absence of a live physiological response means that the early healing and osseointegration processes could not be assessed. This model was selected specifically to provide a standardized, reproducible, and ethically feasible substrate for assessing immediate mechanical outcomes in low-density bone, without the biological variability that accompanies *in vivo* healing models. Therefore, while these results provide strong evidence for enhanced primary mechanical stability, further *in vivo* animal studies and ultimately human clinical trials are necessary to confirm these benefits and evaluate long-term biological outcomes.

## 6 Conclusion

The clockwise osseodensification (OD) shows a trend toward improved primary implant stability in low-density bone. While the findings indicate potential clinical advantages, they do not confirm statistical superiority over standard drilling. Further studies with larger sample sizes and *in vivo* models are required to validate these outcomes. By densifying bone, the technique yielded clinically relevant improvements in stability metrics. More importantly, it established a significant correlation between insertion torque and ISQ. This suggests the simplified OD protocol enhances predictability. While these cadaveric results are encouraging, clinical trials are essential to validate its efficacy and safety in practice.

## Data Availability

The raw data supporting the conclusions of this article will be made available by the authors, without undue reservation.
