# Supplementary material for: Innovation of clockwise osseodensification technique for primary stability in dental implant: a low-density bone cadaveric study
Source: Front Dent Med. 2025 Nov 12;6:1712749. doi: 10.3389/fdmed.2025.1712749 (PMC12647120; doi:10.3389/fdmed.2025.1712749)
Supplement: Supplementary file 1 [file Table1.docx]

# Supplementary File: AI Use Declaration

## 1. Introduction

This supplementary file provides details of the generative AI technology used in preparing the manuscript, in compliance with the Frontiers in Dental Medicine guidelines. The AI was used solely to improve the clarity, grammar, and phrasing of the manuscript. The authors verified all AI-assisted content for factual accuracy, scientific integrity, and originality before inclusion in the final submission.

## 2. AI Technology Used

Tool: ChatGPT
Model: GPT-5
Developer: OpenAI
Access URL: https://chat.openai.com
Date of Access: September 2025

## 3. Prompts Provided to the AI

Example Prompt:
“Please rephrase and polish this section of the manuscript for grammar, clarity, and conciseness while maintaining scientific accuracy.”

## 4. AI Outputs

The outputs from ChatGPT (GPT-5) included rephrased and grammatically corrected text suggestions. Below is an example of AI-assisted output (before and after revision by the authors):

### Example

Original text provided by authors:

Nine formalin-fixed cadaveric tibia bone samples were used. A total of 40 Osstem Taper TS III implants (4.0 mm diameter, 10.0 mm length; Osstem®, Korea) were placed, with 20 sites assigned to the SD group and 20 to the OD group.

AI-assisted rephrased text:

Nine formalin-fixed human tibiae were used. A total of 40 Osstem Taper TS III implants (4.0 mm diameter, 10.0 mm length; Osstem®, Korea) were placed, with 20 sites allocated to the standard drilling (SD) group and 20 to the osseodensification (OD) group.

The final manuscript version incorporated the AI-assisted suggestion after careful review, verification, and editing by the authors.
